# Comparative functional genomic analysis of table olive-associated *Lactiplantibacillus plantarum* strains

**DOI:** 10.3389/fbinf.2026.1936258

**Published:** 2026-09-14

**Authors:** Elio López-García, Antonio Benítez-Cabello, Efstathios Z. Panagou, José Luis Ruiz-Barba, Francisco Noé Arroyo-López

**Affiliations:** 1 Food Biotechnology Department, Instituto de La Grasa (CSIC), Campus Universitario Pablo de Olavide, Seville, Spain; 2 Laboratory of Microbiology and Biotechnology of Foods, Department of Food Science and Human Nutrition, School of Food and Nutritional Sciences, Agricultural University of Athens, Athens, Greece

**Keywords:** comparative genomics, hybrid assembly, lactic acid bacteria, long reads, mobilome

## Abstract

Table olives are among the most important fermented vegetables in the Mediterranean region, with fermentations largely driven by *Lactiplantibacillus* species. While *Lactiplantibacillus pentosus* has been extensively studied at the genomic level, genomic information on olive-associated *Lactiplantibacillus plantarum* remains scarce. To expand current knowledge, three novel *L. plantarum* strains isolated from table olive fermentations in Spain (Lpl15 and LPT703) and Greece (B282) were subjected to whole-genome sequencing and comparative genomic analysis. Genome assemblies ranged from 3.25 to 3.50 Mb and contained 3,060–3,328 predicted protein-coding genes. While Illumina assemblies remained fragmented, hybrid sequencing produced a complete genome assembly for strain *L. plantarum* Lpl15, including a complete chromosome and three plasmids. Comparative analyses incorporating *L. plantarum* genomes from diverse ecological origins, including vegetable fermentations, animal-derived foods, and human-associated environments, were performed using Average Nucleotide Identity (ANI), pangenome analysis, and core-genome phylogeny. Neither ANI nor phylogenomic analyses supported the existence of a distinct lineage associated with table olive fermentations. Functional and metabolic comparisons revealed two major genomic groups linked to ecological origin. Genomes predominantly associated with vegetable fermentations lacked complete nitrogen metabolism pathways, whereas strains of animal origin generally retained these functions. In addition, vegetable-associated strains showed an enrichment of genes involved in exopolysaccharide biosynthesis. Analyses of protein families, domains, and mobilome-related elements further revealed niche-associated genomic patterns. Overall, these results indicate that *L. plantarum* from table olive fermentations does not constitute a distinct evolutionary lineage. Instead, adaptation appears to be reflected in functional and accessory genomic traits associated with ecological origin, providing new insights into the diversity and adaptive potential of this species across different environments.

## 1 Introduction

Table olives are among the most widely consumed fermented vegetables in the Mediterranean region, with global production exceeding 3 million tonnes during the 2024/2025 season (IOC, 2026). Their fermentation is a complex microbial process dominated by species of the genus *Lactiplantibacillus*, particularly *L. pentosus* and *L. plantarum* (Hurtado et al., 2011). These bacteria contribute not only to acidification and product stability but also to sensory quality, food safety, and the functional properties of fermented olives (Randazzo et al., 2014; Benítez-Cabello et al., 2020). Most genomic studies of table olive fermentations have focused on *L. pentosus*, for which more than 40 genomes sequenced from olive origin are currently available in public databases. These studies have provided valuable insights into its genetic diversity, adaptation to the fermentation environment, and technological potential, supporting the optimization of fermentation processes and product quality (Abriouel et al., 2017; López-García et al., 2023a).

In contrast, genomic studies of *L. plantarum* in table olive fermentations remain scarce, despite the ecological and technological relevance of this species. To date, only four complete genomes from olive-associated *L. plantarum* strains are publicly available, limiting our understanding of their genetic diversity, adaptive traits, and functional potential in this fermentation ecosystem. Given its remarkable ecological versatility and occurrence across a wide range of fermented foods, gastrointestinal environments, and natural habitats, genomic characterization of table olive-derived strains is essential to elucidate the mechanisms underlying their adaptation and contribution to the fermentation process.

Advances in Next-Generation Sequencing (NGS) technologies have transformed microbial genomics by enabling the rapid generation of high-resolution genome sequences. Among these approaches, Whole Genome Sequencing (WGS) provides comprehensive insights into genome organization, metabolic potential, adaptive mechanisms, and other functionally relevant traits (Tenea and Ortega, 2021; Raethong et al., 2022). Such information is fundamental for understanding microbial ecology and for the development of strategies aimed at improving traditional fermentation processes.

In this study, three novel *L. plantarum* strains isolated from table olive fermentations in Spain and Greece were subjected to whole-genome sequencing and comparative genomic analysis. The results expand the currently limited genomic information available for olive-associated *L. plantarum* and provide insights into its genetic diversity, functional and technological capabilities, and adaptation to the fermentation environment. Comparative analyses with publicly available genomes enabled the identification of genetic traits potentially linked to ecological fitness and technological performance. These findings enhance our understanding of the role of *L. plantarum* in table olive fermentations and support the future development of strains with improved biotechnological and probiotic applications.

## 2 Materials and methods

### 2.1 Bacterial isolation, DNA extraction, and genome sequencing

The 3 *L. plantarum* strains used in this study (Lpl15, B282, and LPT703) were isolated from table olive fermentations, plating on de Man–Rogosa–Sharpe (MRS) agar supplemented with 0.02% (w/v) sodium azide (Sigma-Aldrich, St. Louis, MO, United States) and incubated at 37 °C for 48 h. *Lactiplantibacillus plantarum* B282 was isolated from natural black olive fermentation in Greece, whereas *L. plantarum* Lpl15 and LPT703 were recovered from Spanish-style (lye-treated) green olive fermentations. Preliminary identification was performed by 16S rRNA gene sequencing, and species-level assignment within the *L. plantarum* group was confirmed by multiplex PCR targeting the recA gene (Torriani et al., 2001). Before DNA extraction, strains were cultivated in MRS broth (Oxoid, Basingstoke, United Kingdom) at 37 °C for 48 h. Genomic DNA was extracted and purified according to the protocol described by Martín-Platero et al. (2007). The strains B282 and LPT703 were sequenced on an Illumina MiSeq platform (FISABIO, Valencia, Spain) using the Nextera DNA Flex Library Prep Kit (Illumina, San Diego, CA, United States), generating paired-end reads of 2 × 250 bp. On the contrary, the strain Lpl15 was sequenced on an Illumina NextSeq 500 platform (Eurofins, Logroño, Spain) using the same library preparation kit, generating paired-end reads of 2 × 150 bp. In addition, Lpl15 was also subjected to long-read sequencing using Oxford Nanopore Technologies (ONT) on a MinION Mk1D device (Instituto de la Grasa, Seville, Spain). Libraries were prepared using the Ligation Sequencing Kit V14 (SQK-LSK114) and sequenced on R10.4.1 flow cells according to the manufacturer’s instructions. Nanopore reads were basecalled using the super-accurate model. Illumina reads were quality-filtered and adapter-trimmed using TrimGalore v0.6.10 (Krueger, 2021), whereas ONT reads were processed with Porechop v0.2.4 (Wick, 2017a). Read quality was assessed using FastQC v0.11.9 (Andrews, 2010) for Illumina data and NanoPlot v1.44.1 (De Coster et al., 2018) for ONT data. Raw sequencing reads and annotated genome assemblies have been deposited in the European Nucleotide Archive (ENA) under BioProject accession number PRJEB111681.

### 2.2 Genome assembly, quality assessment, synteny analysis, and annotation

Genome assemblies were generated using SPAdes v4.2.0 (Bankevich et al., 2012) with a minimum contig length threshold of 300 bp. For strain Lpl15, a hybrid assembly combining Illumina short reads and Oxford Nanopore long reads was performed using Unicycler v0.5.1 (Wick et al., 2017b), yielding a complete circularized genome. Assembly quality was evaluated by comparing standard assembly metrics, including the number of contigs, longest contig length, total genome size, N50 value, and GC content. Genome completeness and contamination were also assessed using CheckM v1.2.2 (Parks et al., 2015). Futhermore, genome completeness was assessed using BUSCO v5.8.0 (Simão et al., 2015) with the bacteria_odb10 and lactobacillales_odb10 lineage datasets, which contain conserved single-copy orthologs representative of Bacteria and the order *Lactobacillales*, respectively. Genome synteny was evaluated by aligning the draft genome assemblies of strains B282 and LPT703 against the complete hybrid genome assembly of strain Lpl15 using MUMmer v4.0.0 (Marçais et al., 2018). The structural accuracy of the Lpl15 assembly was further assessed by comparison with the complete genome of *L. plantarum* SRCM100442. Structural genome annotation was performed using Prokka v1.14.16 (Seemann, 2014), while rRNA genes were identified using Barrnap v1.10.5 (Seemann, 2013). Functional annotation was conducted using eggNOG-mapper v5.0 (Huerta-Cepas et al., 2019), allowing the assignment of genes to Clusters of Orthologous Groups (COG) functional categories. Putative bacteriocin and ribosomally synthesized and post-translationally modified peptide (RiPP) gene clusters were identified using BAGEL5 (Van Heel et al., 2018). Exopolysaccharide (EPS) biosynthesis clusters were manually curated by BLASTp searches against reference protein sequences, applying thresholds of 50% amino acid identity, 70% query coverage, and an E-value ≤1 × 10^−9^.

### 2.3 Defense systems and mobilome characterization

Defense mechanisms against exogenous DNA were investigated using several bioinformatic approaches. CRISPR arrays and CRISPR-associated (cas) genes were identified using CRISPRCasFinder v1.1.2-I2BC (Grissa et al., 2007), and predicted CRISPR loci were validated against the CRISPRdb database. In addition, DefenseFinder v2.0.0 (Tesson et al., 2024) was employed to identify known bacterial anti-phage and anti-mobile genetic element defense systems. The mobilome of the 3 *L. plantarum* isolates was characterized through the prediction of major mobile genetic elements (MGEs), including plasmids, genomic islands (GIs), insertion sequences (ISs), integrons, and prophages. Putative plasmid-derived contigs were identified using RFPlasmid v1.0 (van der Graaf-Van Bloois et al., 2021). Predicted plasmid sequences were subsequently compared with plasmids from phylogenetically related strains using BLAST against the NCBI database. GIs were identified using IslandViewer4 (Bertelli et al., 2017), whereas integrons were detected using IntegronFinder v2.0.5 (Néron et al., 2022). Prophage regions were predicted with PHASTEST (Wishart et al., 2023), and ISs elements were identified using ISEScan v1.7.3 (Xie and Tang, 2017).

### 2.4 Genomic safety assessment

The genomic safety of the 3 *L. plantarum* isolates was evaluated using multiple bioinformatic tools. Putative antimicrobial resistance determinants were identified using the Comprehensive Antibiotic Resistance Database (CARD) together with the Resistance Gene Identifier (RGI) pipeline (Alcock et al., 2020). Acquired antimicrobial resistance (AMR) genes were additionally screened using ResFinder v4.0 (Bortolaia et al., 2020) with thresholds of 80% sequence identity and 60% minimum gene coverage. Potential pathogenicity was assessed using PathogenFinder v1.1 (Cosentino et al., 2013), while acquired virulence-associated genes were investigated using VirulenceFinder v2.0 (Joensen et al., 2014). The combined results were used to evaluate the safety profile of the isolates and their suitability for potential technological applications.

### 2.5 Taxonomic identification and phylogenomic analysis

To assess the genomic diversity of the newly sequenced isolates, a comparative dataset comprising 35 *L. plantarum* genomes was assembled, including the three strains generated in this study and 32 publicly available genomes retrieved from NCBI GenBank (accessed on 4 Ma y 2026) (see Supplementary Table S1, Supplementary Material). The selected strains came from diverse ecological niches, including vegetable fermentations (table olives, kimchi, sauerkraut, and pickles), dairy products, meat-derived foods, and human-associated microbiota. Average Nucleotide Identity (ANI) analysis was performed using a Python script (Pritchard et al., 2014). The resulting similarity matrix was visualized by hierarchical clustering and heatmap representation using the pheatmap package in R software. Before comparative analyses, all genomes were reannotated using Prokka to minimize annotation-related biases. Pangenome analysis was conducted using Panaroo v1.2.9 (Tonkin-Hill et al., 2020) with a 95% BLASTp identity threshold. The pangenome was partitioned into core and accessory gene clusters, using a prevalence threshold of 95% across genomes. A core-genome phylogeny was reconstructed using IQ-TREE2 (Minh et al., 2020) with 1,000 bootstrap replicates and visualized with iTOL (Letunic and Bork, 2024).

### 2.6 Comparative metabolic and functional genomic analyses

To investigate potential genomic adaptations associated with ecological origin, genes were assigned to Kyoto Encyclopedia of Genes and Genomes (KEGG) Orthology (KO) (Kanehisa et al., 2016) categories. KO annotations were subsequently analyzed using KEGGDecoder v.1.3 (Graham et al., 2018) to estimate metabolic pathway completeness across genomes. Metabolic profiles were subsequently used for hierarchical clustering. Given the diversity of carbohydrate substrates encountered by *L. plantarum* across different ecological niches, Carbohydrate-active enzymes (CAZymes) were predicted using dbCAN (Zheng et al., 2023), and presence–absence profiles were visualized by hierarchical clustering and heatmap analysis. Based on the dendrogram topology, genomes were partitioned into three major clusters. Differences in CAZy family prevalence among clusters were evaluated using Fisher’s exact test with Benjamini–Hochberg false discovery rate (FDR) correction. To further investigate niche-associated genomic traits, protein families and domains were annotated using the Pfam database (Paysan-Lafosse et al., 2025). Highly variable Pfam domains were retained for downstream analyses and explored by Principal Component Analysis (PCA). Subsequently, a Random Forest analysis was conducted using the R package randomForest v.4.7 (Svetnik et al., 2003) with 500 trees, and variable importance scores were extracted to identify the Pfam domains most strongly associated with ecological origin.

## 3 Results

### 3.1 Sequencing and genome assembly comparison

Sequencing statistics for the 3 *L. plantarum* isolates are summarized in Table 1. Illumina sequencing generated high-quality datasets for all strains, while Oxford Nanopore sequencing of strain Lpl15 provided long reads with an average length of 10.8 kb, substantially exceeding the read lengths obtained with Illumina platforms (<210 bp). Following quality filtering, all datasets achieved approximately 100X genome coverage. Genome assembly results are summarized in Table 2. Draft assemblies generated from Illumina short reads contained 42 and 22 contigs for strains B282 and LPT703, respectively. In contrast, hybrid assembly of strain Lpl15 using Illumina and Oxford Nanopore reads resulted in a highly contiguous assembly comprising four contigs. Genome sizes ranged from 3.20 to 3.45 Mb, with GC contents between 44.17% and 44.43%. BUSCO analysis confirmed high assembly completeness, recovering all conserved bacterial (124/124) and *Lactobacillales* (402/402) orthologs without fragmented or missing genes (Table 3). Consistent with the BUSCO results, CheckM analysis confirmed the high quality of three genome assemblies, with high completeness and low contamination values (Supplementary Table S2). Genome alignments revealed overall chromosomal synteny among strains while highlighting the greater fragmentation of short-read assemblies (Figure 1).

**TABLE 1:** Sequencing statistics, read processing, and genome coverage obtained for the 3 *L. plantarum* strains using Illumina and Oxford Nanopore sequencing platforms.

Strain	Sequencing platform	Raw reads number	Mean raw read length (bp)	Trimmed and normalized reads number	Mean processed reads length (bp)	Total bases (Gb)	Estimated genome coverage (x)
B282	Illumina	1,701,076	113.3	1,508,778	114.5	0.345	104.26
Lpl15	Illumina	41,690,940	149.5	6,253,638	149.5	0.934	273.72
	ONT	229,823	5,097.3	55,603	10,790.8	0.6	174.93
LPT703	Illumina	1,641,200	204.9	1,461,736	208.2	0.304	94.77

**TABLE 2:** Assembly statistics of the 3 *L. plantarum* genomes generated using short-read (B282 and LPT703) and hybrid (Lpl15) assembly approaches.

Strain	Assembler	Contigs	Length of the longest contig (bp)	N50 (bp)	L50	Genome lenght (bp)	G/C (%)
B282	SPAdes	42	493,106	270,699	5	3,447,238	44.17
Lpl15	Unicycler	4	3,274,892	3,274,892	1	3,405,362	44.31
LPT703	SPAdes	22	640,657	372,704	4	3,200,160	44.43

**TABLE 3:** BUSCO assessment of genome completeness based on bacterial (bacteria_odb10) and *Lactobacillales*-specific (*lactobacillales*_odb10) conserved orthologs.

	BUSCO bacteria_odb10				BUSCO *lactobacillales*_odb10			
Strain	C	D	F	M	C	D	F	M
B282	124/124	1	0	0	402/402	2	0	0
Lpl15	124/124	1	0	0	402/402	2	0	0
LPT703	124/124	1	0	0	402/402	3	0	0

C: Complete; D: Duplicated; F: Fragmented; M: Missing.

**FIGURE 1:**
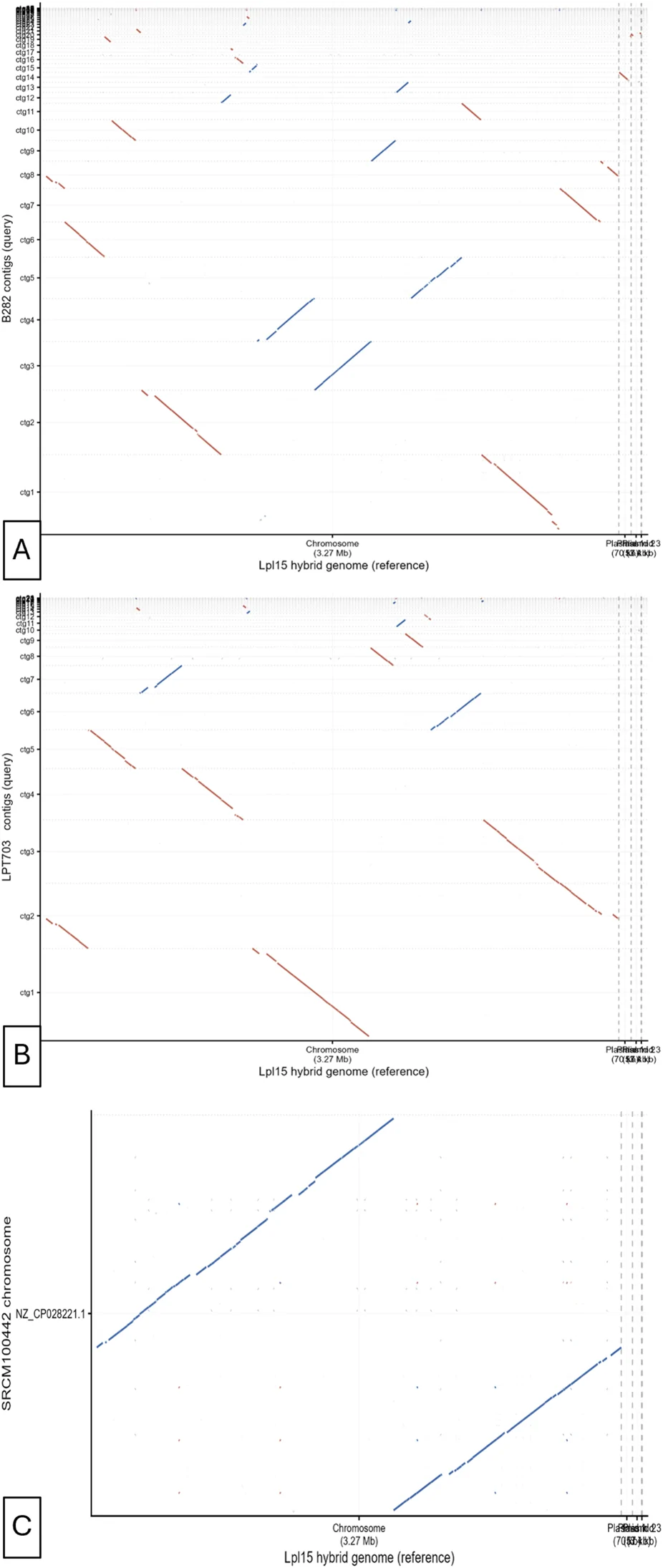
Genome synteny analysis of *L. plantarum* strains. Dot plots comparing the assemblies of strains B282 (red) and Lpl15 (blue) **(A)**, LPT703 (red) and Lpl15 (blue) **(B)**, and Lpl15 (blue) and the reference strain *L. plantarum* SRCM100442 (red) **(C)**.

Prokka annotation predicted 3,328, 3,282, and 3,060 coding sequences (CDS) in strains B282, Lpl15, and LPT703, respectively (Table 4). Three plasmids were identified in the hybrid assembly of Lpl15, whereas plasmid assignment was not possible for the fragmented assemblies of B282 and LPT703. Predicted rRNA genes differed markedly among strains, with 8, 16, and 2 genes detected in B282, Lpl15, and LPT703, respectively. Functional annotation showed that transcription, translation, and carbohydrate transport and metabolism were the most represented COG categories, whereas cell motility, secondary metabolite biosynthesis, and cell cycle control were the least abundant (Figure 2). BAGEL5 predicted a single class II bacteriocin cluster corresponding to plantaricin J in all three genomes (see Supplementary Table S3, Supplementary Material). Analysis of exopolysaccharide (EPS) loci identified two complete EPS biosynthesis clusters in strains B282 and LPT703, whereas strain Lpl15 contained a single cluster with the same genetic organization as one of the loci present in the other two strains (see Supplementary Table S4, Supplementary Material).

**TABLE 4:** Summary of genome annotation statistics, including predicted coding sequences, rRNA genes, and plasmid identification.

Strain	Annotator	Number of CDS	Number of plasmids	Number of rRNA genes
B282	Prokka + barnap	3,328	NA	8
Lpl15	Prokka + barnap	3,282	3	16
LPT703	Prokka + barnap	3,060	NA	2

**FIGURE 2:**
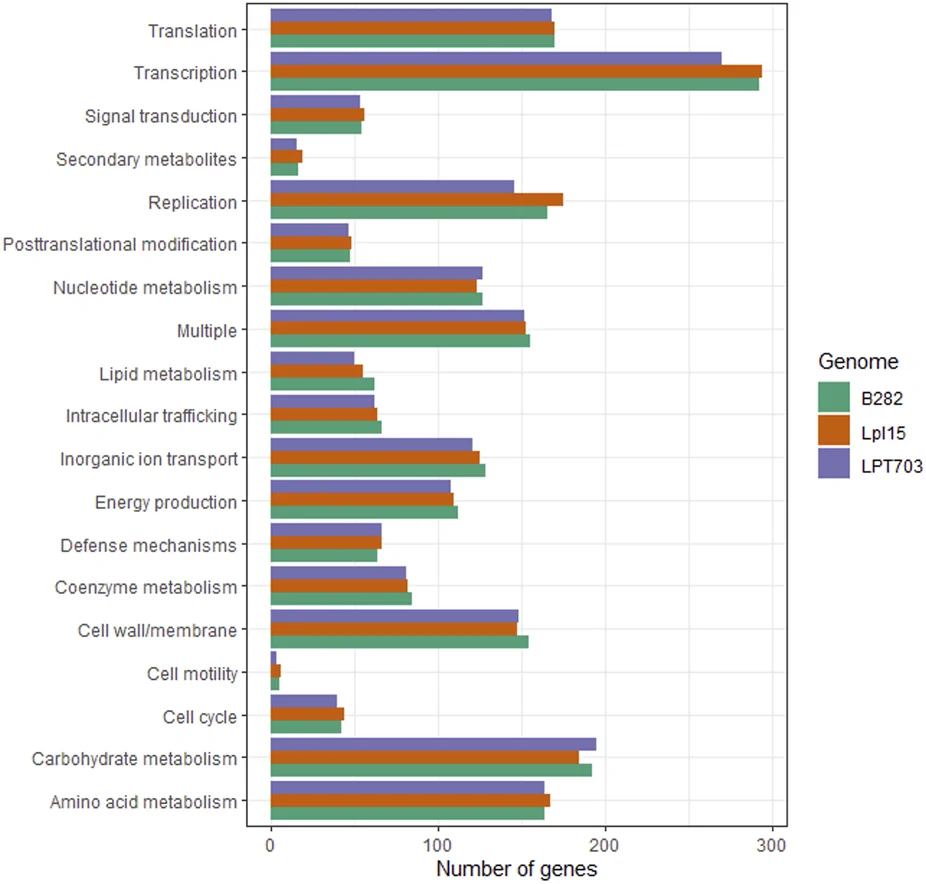
Functional distribution of predicted coding sequences in the 3 *L. plantarum* genomes according to COG (Clusters of Orthologous Groups) categories assigned by eggNOG-mapper. Genes lacking COG annotation or assigned to unknown functions were excluded.

### 3.2 Defense mechanisms and mobilome analysis

CRISPR-Cas systems were largely absent from the 3 *L. plantarum* genomes. Only strain B282 contained an incomplete subtype I *cas* cluster, whereas no *cas* genes were detected in strains Lpl15 and LPT703. Although a putative CRISPR array was predicted in each genome, all predictions showed low confidence. Alternative defense mechanisms were identified in all strains, including AbiD, BREX, CBASS, MazEF, restriction–modification (RM), and Septu systems (Table 5). Strain B282 exhibited the most diverse defensive repertoire, with five distinct defense systems, whereas strain Lpl15 contained only a single predicted defense system. Strain LPT703 displayed an intermediate profile, harboring three different defense mechanisms.

**TABLE 5:** Defense systems identified in the genomes of the 3 *L. plantarum* strains.

	Defense system						
Type	AbiD	BREX	CBASS	MazEF	RM		Septu
Subtype	AbiD	BREX I	CBASS_II	MazEF	RM type I	RM type IV	Septu
B282	-	+ (6)	+ (3)	+ (2)	-	+ (1)	+ (2)
Lpl15	+ (1)	-	-	-	-	-	-
LPT703	+ (1)	-	-	-	+ (3)	+ (1)	-

Number in parenthesis corresponding to the number of genes implicated in the defense mechanisms.

The mobilome composition of the three genomes is summarized in Table 6. Due to the fragmented nature of the short-read assemblies, the number of plasmids and GIs could not be reliably determined for strains B282 and LPT703. In contrast, the complete hybrid assembly of strain Lpl15 revealed three plasmids and three GIs. The plasmids measured 69,974 bp, 57,072 bp, and 3,424 bp in length. The closest plasmid matches identified by BLAST searches against the NCBI plasmid database are listed in Table 7. Most alignments covered more than 60% of the reference plasmid sequence, and all exhibited nucleotide identities above 97%. No integrons were detected in any of the three genomes. Predicted ISs ranged from five to seven elements in the short-read assemblies, whereas 16 ISs were identified in the hybrid assembly of strain Lpl15. Similarly, three intact prophages were detected in strains B282 and Lpl15, while only one intact prophage was identified in strain LPT703.

**TABLE 6:** Summary of mobile genetic elements identified in the genomes of the 3 *L. plantarum* strains.

Strain	Number of plasmids	Number of genomic islands	Number of integrons	Number of insertion sequences	Number of prophages
B282	NA	NA	0	5	3
Lpl15	3	3	0	16	3
LPT703	NA	NA	0	7	1

**TABLE 7:** Closest plasmid matches identified by BLAST analysis of the three plasmids detected in *L. plantarum* Lpl15.

seq.ID	Plasmid size (bp)	Genes number	Accession number	Organism/Strain	Query coverage (%)	Identity (%)
Lpl15_contig_2	69,974	66	CP013750.1	*L. plantarum* strain KP plasmid unnamed1	93	99.72
Lpl15_contig_3	57,072	56	CP023175.1	*L. plantarum strain BDGP2 plasmid pLtBDGP2AP*	64	97.73
Lpl15_contig_4	3,424	3	JN084215.1	*Lentilactobacillus buchneri* CD034 plasmid pCD034-1	100	99.41

### 3.3 Genomic safety assessment

No acquired antibiotic resistance, disinfectant resistance, virulence, or pathogenicity-associated genes were detected in any genome. Although CARD identified vancomycin resistance determinants, these corresponded to the intrinsic resistance commonly found in lactobacilli. Overall, the results support the genomic safety of the 3 *L. plantarum* isolates sequenced.

### 3.4 ANI, pangenome analysis, and phylogenomic relationships

ANI analysis of the 35 *L. plantarum* genomes confirmed their taxonomic assignment to the species, with pairwise nucleotide identities ranging from 98.25% to 99.99% (Supplementary Figure S1, Supplementary Material). The highest average ANI values were observed between strains isolated from pickles and sauerkraut (99.43%), whereas the lowest were detected between sauerkraut- and dairy-associated strains (98.70%).

Pangenome analysis identified a total of 6,480 gene clusters, comprising 2,351 core gene clusters and 4,129 accessory gene clusters. Phylogenomic reconstruction based on the core-genome alignment generated a maximum-likelihood tree with a predominantly intermixed distribution of strains from different ecological origins (Figure 3). No clear clustering according to isolation source was observed for most genomes. However, strains isolated from cucumber and sauerkraut fermentations formed a distinct lineage that was clearly separated from the remaining *L. plantarum* genomes.

**FIGURE 3:**
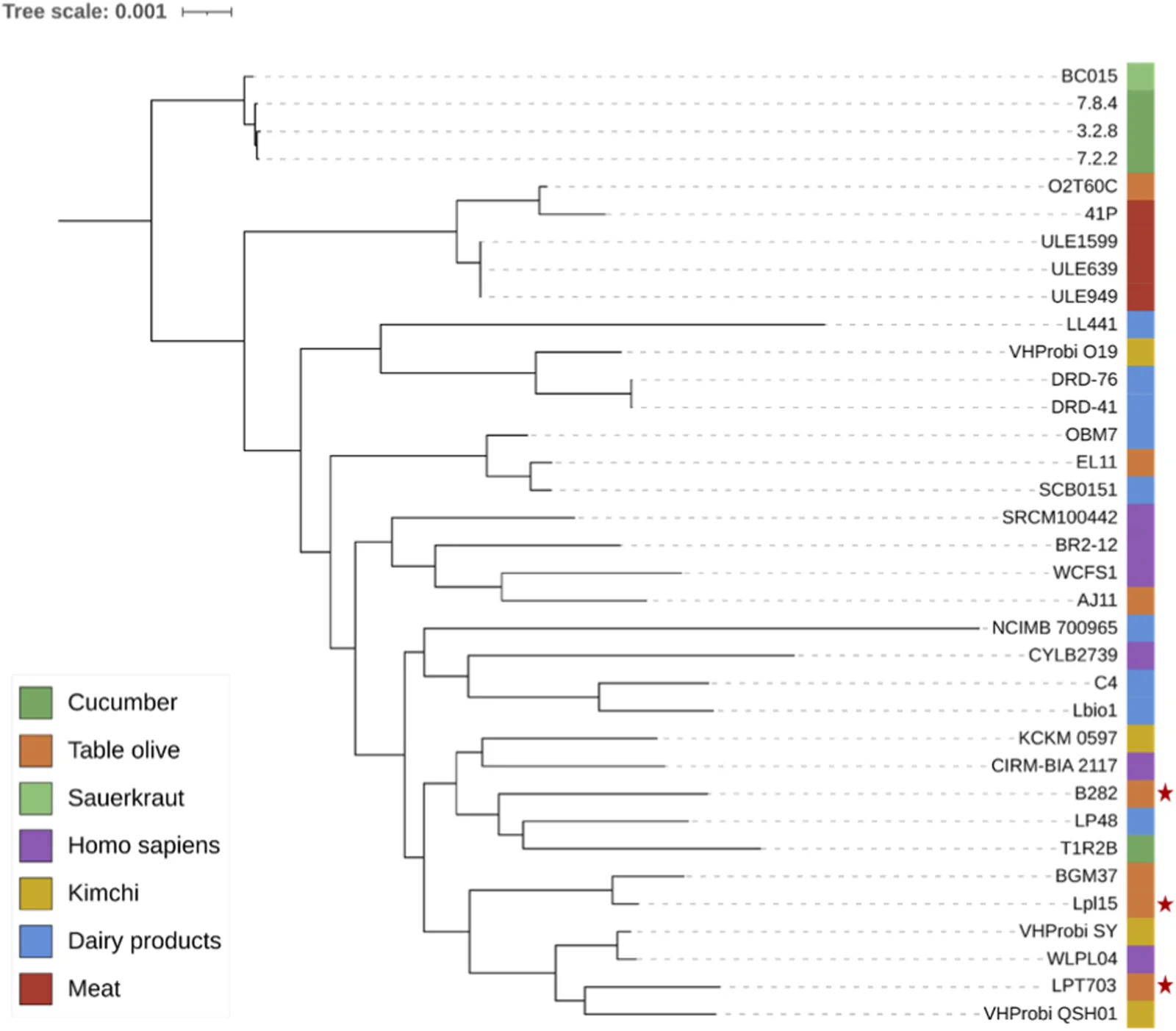
Core-genome phylogenomic tree of 35 *L. plantarum* strains inferred from the alignment of core gene clusters identified in the pangenome. Strain origins are indicated by colored labels: cucumber fermentation (green), table olive fermentation (orange), sauerkraut (light green), human microbiota (purple), kimchi (yellow), dairy products (blue), and meat products (red). The three strains sequenced in this study are marked with red stars.

### 3.5 Comparative metabolic pathway and CAZyme analyses across ecological origins

KEGG analysis identified 62 metabolic pathways, of which 35 were complete in all genomes, whereas 27 displayed variable levels of completeness (Supplementary Table S5, Supplementary Material). Hierarchical clustering based on pathway completeness revealed two major genome groups, as illustrated in Figure 4.

**FIGURE 4:**
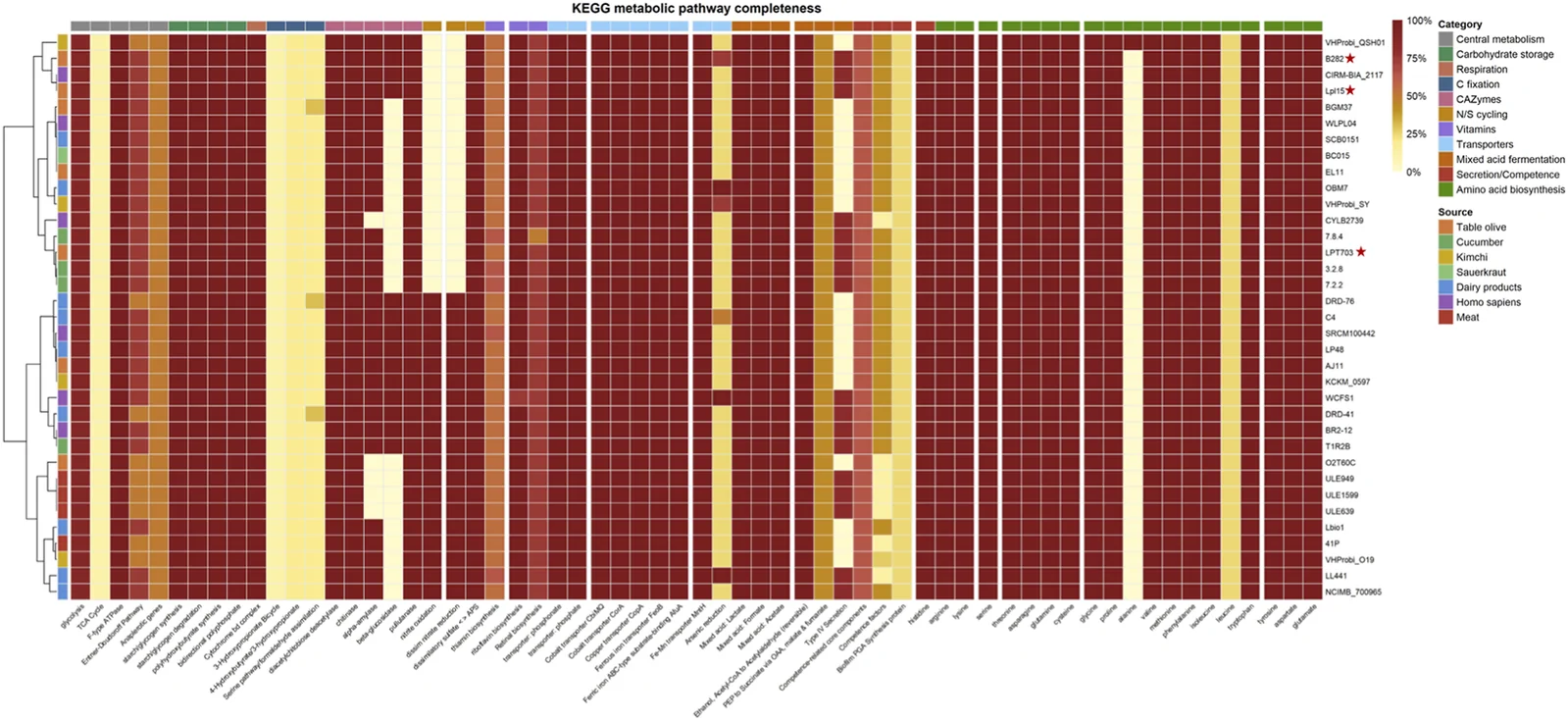
Heatmap showing metabolic pathway completeness across 35 *L. plantarum* genomes based on KEGG annotations. Pathway completeness is represented by a color gradient ranging from light yellow (incomplete) to dark red (complete). The three strains sequenced in this study are marked with red stars.

A total of 57 CAZy families were identified, including 28 glycoside hydrolase (GH), 19 glycosyltransferase (GT), 4 carbohydrate esterase (CE), 3 carbohydrate-binding module (CBM), and 3 auxiliary activity (AA) families. Thirty-one families were present in all genomes. The most abundant CAZy families were GH1, GT2, and GT4, with average copy numbers of approximately 9, 11, and 12 copies per genome, respectively (Supplementary Table S6, Supplementary Material). Hierarchical clustering based on CAZyme profiles grouped the genomes into three major clusters using the R function hclust (Supplementary Table S7), associated predominantly with plant-associated, animal-associated, or mixed ecological origins (Figure 5). Differential prevalence analysis identified GT125, GH31_15, GH20, GH92, and GH125 as significantly enriched in specific clusters (Figure 6).

**FIGURE 5:**
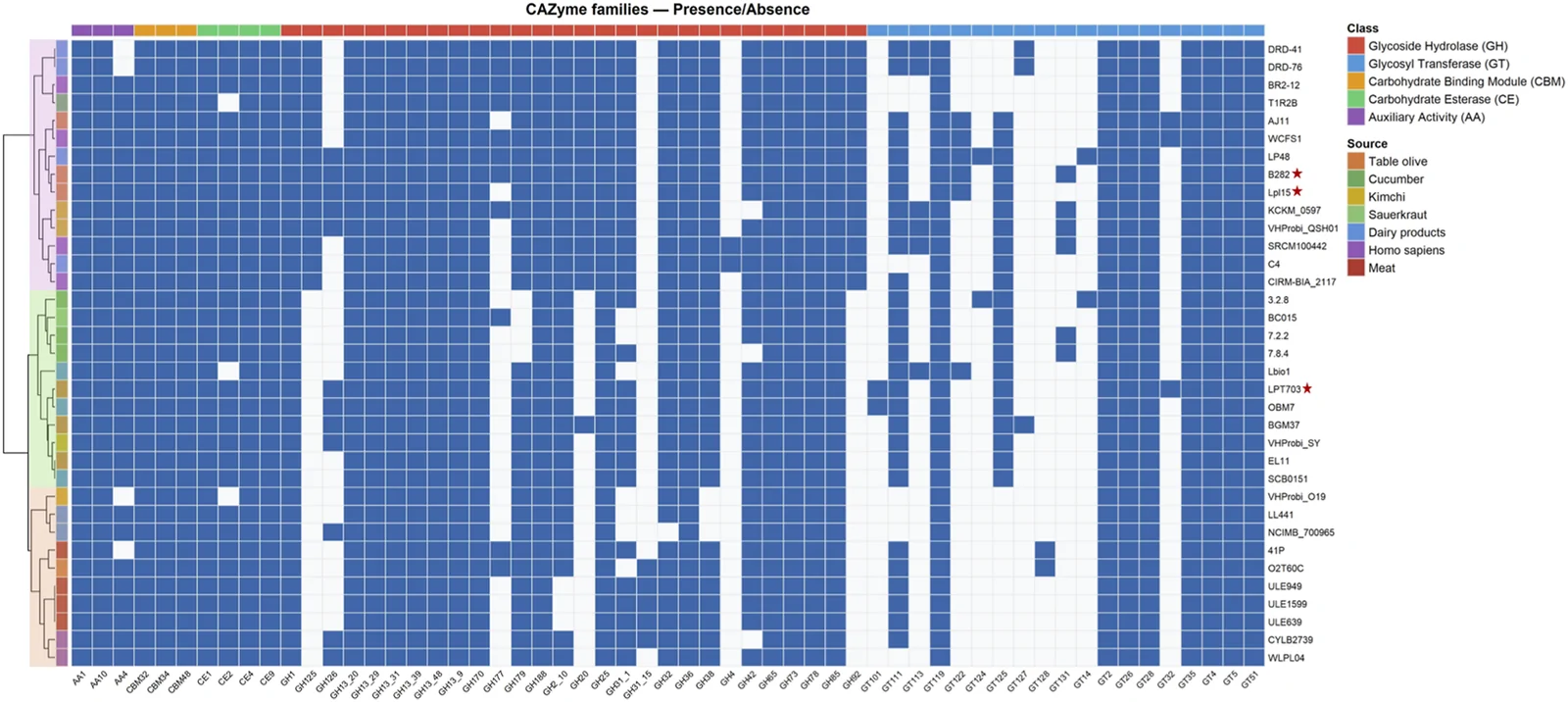
Heatmap showing the presence and absence of CAZyme families across 35 *L. plantarum* genomes. Presence is indicated by filled cells and absence by empty cells. Hierarchical clustering based on CAZyme profiles identified three major genome clusters: Cluster 1 (predominantly plant-associated, green), Cluster 2 (predominantly animal-associated, orange), and Cluster 3 (mixed ecological origins, purple). The three strains sequenced in this study are marked with red stars.

**FIGURE 6:**
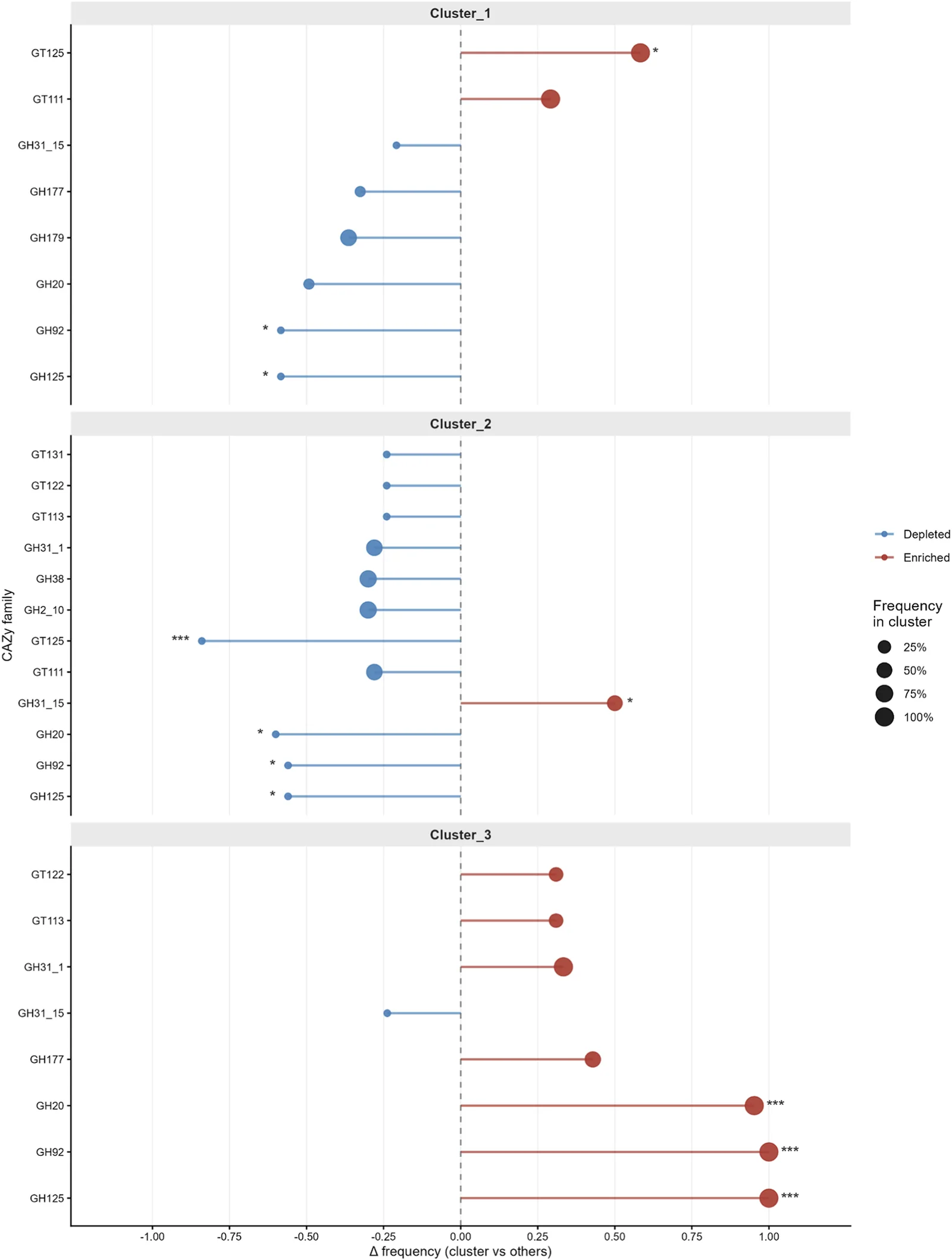
Differential enrichment of CAZyme families among the three genome clusters identified in Figure 5: Cluster 1 (predominantly plant-associated), Cluster 2 (predominantly animal-associated), and Cluster 3 (mixed ecological origins). Statistical significance was assessed using Fisher’s exact test followed by Benjamini–Hochberg false discovery rate (FDR) correction.

Pfam annotation identified a total of 2,284 protein families and domains across the 35 *L. plantarum* genomes. After variance-based filtering, 300 domains were retained for downstream analyses. Principal Component Analysis (PCA) showed that the first two principal components explained approximately 30% of the total variance among genomes from different ecological origins (Figure 7). Variable importance analysis identified the Pfam domains that contributed most to the discrimination of ecological origin (Supplementary Table S8). Functional classification of these domains revealed one domain related to adhesion, five domains of unknown function (DUFs), six domains associated with defense systems, three domains linked to mobile genetic elements, three prophage-related domains, one DNA regulatory domain, and two restriction–modification-associated domains. The distribution of the most informative Pfam categories across ecological origins is show in Figure 8.

**FIGURE 7:**
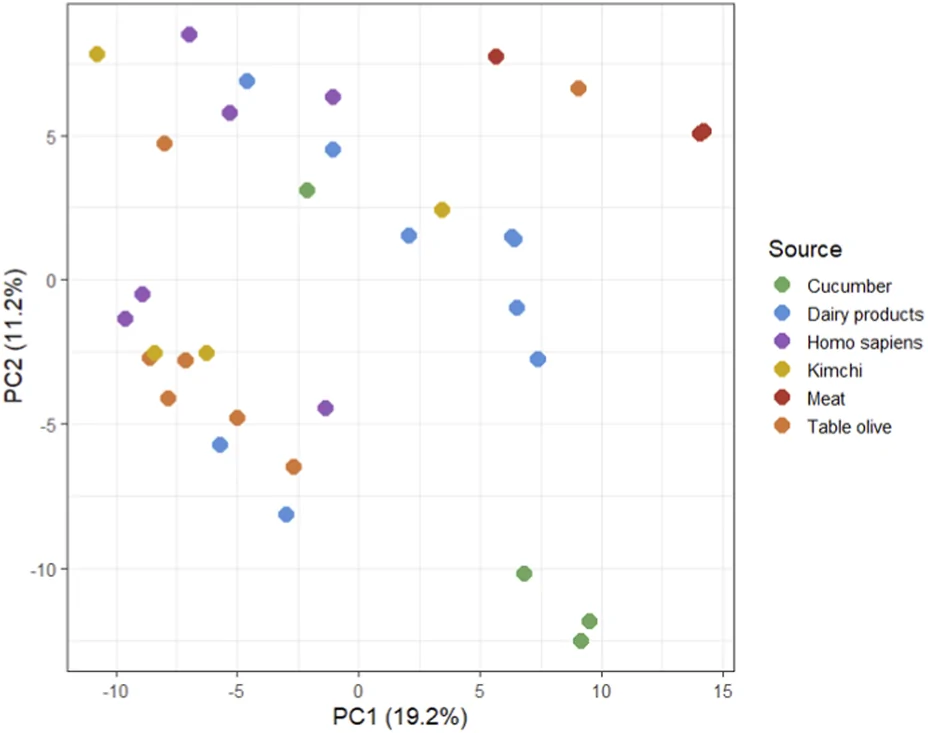
Principal component analysis (PCA) of the 35 *L. plantarum* genomes based on Pfam protein family and domain profiles. Genomes are colored according to their ecological origin.

**FIGURE 8:**
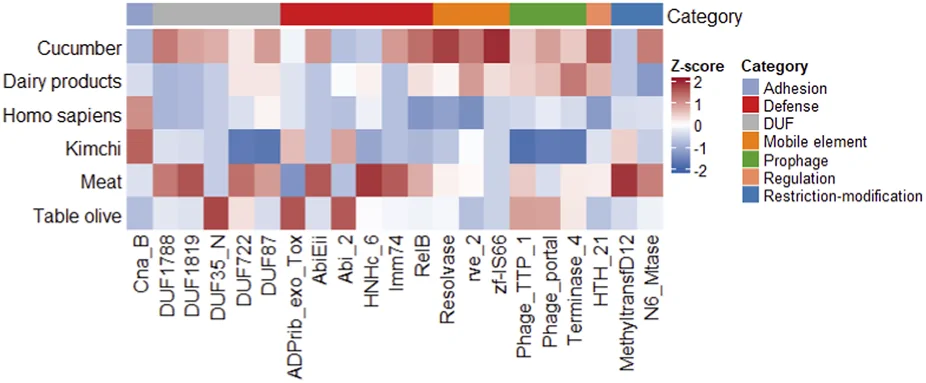
Distribution of the most informative Pfam functional categories across the ecological origins of *L. plantarum* strains.

## 4 Discussion

The sequencing strategies employed in this study produced assemblies of markedly different contiguity. While the Illumina-based assemblies of strains B282 and LPT703 remained fragmented (42 and 22 contigs, respectively), the hybrid assembly of strain Lpl15 yielded a complete chromosome and three plasmids. Similar observations were recently reported by Marín-Gordillo et al. (2026), who compared Illumina-, ONT-, and PacBio-based assemblies in *Lactiplantibacillus pentosus*, a close relative species of *L. plantarum*. Their study demonstrated that short-read assemblies frequently remain fragmented due to the inability of short reads to resolve repetitive genomic regions, whereas long-read and hybrid approaches enable chromosome closure and improved characterization of plasmid content. Despite the higher fragmentation observed in the Illumina-only assemblies, genome sizes ranged from 3.20 to 3.45 Mb, which is consistent with the expected genome size reported for *L. plantarum*. Moreover, BUSCO analyses recovered the complete set of conserved bacterial and *Lactobacillales* orthologs in all assemblies, indicating that the genomes were nearly complete despite remaining fragmented. These results highlight an important distinction between genome completeness and assembly contiguity: draft assemblies may contain most of the genomic information while still lacking the structural resolution required for chromosome closure. The limitations of short-read assemblies became particularly evident during synteny analyses (Piot et al., 2026). Alignment of strains B282 and LPT703 against the complete Lpl15 genome revealed extensive assembly fragmentation, complicating the reconstruction of chromosomal organization and large-scale structural features. In contrast, alignment of the complete Lpl15 genome with the reference strain *L. plantarum* SRCM100442 showed a high degree of synteny across the chromosome, although a large chromosomal rearrangement was observed relative to the reference genome. Such structural variation has previously been reported within the *Lactiplantibacillus* genus and may reflect the genomic plasticity that characterizes this species (Marín-Gordillo et al., 2026). Genome annotation identified between 3,060 and 3,328 CDS across the three isolates, with larger genomes containing a greater number of predicted genes. Another notable observation was the lower number of predicted rRNA genes in assemblies generated exclusively from Illumina reads compared with the hybrid assembly. This limitation is expected because repetitive rRNA genes are difficult to resolve using short-read data alone, often resulting in collapsed or incomplete assemblies of these regions (Zhang et al., 2021). Consequently, hybrid assemblies provide a more accurate representation of repetitive genomic features, including rRNA genes and plasmid sequences.

Functional annotation based on COG classification revealed that the most represented functional categories in the 3 *L. plantarum* genomes were transcription, translation, and carbohydrate transport and metabolism. Similar distributions have previously been reported for both *L. plantarum* and *L. pentosus*, suggesting a high degree of genomic conservation among these species and reflecting their strong capacity to regulate gene expression and efficiently exploit a wide range of carbohydrate substrates. In contrast, the category cell motility contained the lowest number of predicted genes. These findings are consistent with previous genomic studies of *L. plantarum* and reflect the biological characteristics of the species (Peng et al., 2021; Tenea and Ortega, 2021). In silico analysis identified bacteriocin-associated gene clusters in all 3 *L. plantarum* genomes. The class II bacteriocin plantaricin EF was present in all strains, while plantaricin JK and plantaricin A loci were detected in strains B282 and Lpl15. In contrast, strain LPT703 harbored a plantaricin NO locus. The occurrence of plantaricin-related loci is common within *L. plantarum* populations and has been reported in numerous strains isolated from fermented foods and plant-associated environments (Anderssen et al., 1998). Plantaricins are among the best-characterized bacteriocins produced by *L. plantarum* and are thought to contribute to ecological competitiveness by inhibiting closely related Gram-positive bacteria and other LAB during fermentation processes (Anderssen et al., 1998). Previously, Maldonado-Barragán et al. (2013) identified the production of plantaricin EF and JK in the LPT703 strain. Another study showed that Lpl15 strain had an antimicrobial effect against *Escherichia coli* and *Listeria monocytogenes* (Benítez-Cabello et al., 2019). However, this antimicrobial effect was attributed to lactic acid production rather than bacteriocin activity. On the contrary, Argyri et al. (2013) did not observe an antimicrobial effect of B282 against *E. coli, Salmonella Enteritidis*, or *L. monocytogenes*. The ocurrence the plantaricin in all three isolates suggests that these strains retain the genetic potential to participate in microbial competition and community structuring within table olive fermentations (Maldonado-Barragán et al., 2013; Hurtado et al., 2011).

EPS biosynthesis is another trait of particular interest in *L. plantarum* due to its potential contribution to stress tolerance, biofilm formation, host interactions, and the technological properties of fermented foods (Dertli et al., 2015). In lactobacilli, heteropolysaccharide EPS clusters are typically synthesized through the Wzy-dependent pathway and generally exhibit a conserved organization, with regulatory genes located at the 5′region, glycosyltransferases in the central region, and polymerization/export genes at the 3′end (Deo et al., 2019). According to Deo et al. (2019), functional EPS loci typically contain genes encoding a priming glycosyltransferase, multiple glycosyltransferases, the w*zx* flippase gene, the w*zy* polymerase gene, and a phosphoregulatory module composed of a protein tyrosine kinase and its cognate modulator. The EPS cluster shared by strains B282, Lpl15, and LPT703 exhibited a genomic organization highly similar to the *cps4* locus described in *L. plantarum* WCFS1 (Remus et al., 2012). The presence of genes involved in activated sugar precursor biosynthesis, including a UDP-N-acetylglucosamine 4-epimerase, further suggests the capacity to produce structurally complex heteropolysaccharides (Schmid et al., 2015). Interestingly, strains B282 and LPT703 harbored a second complete EPS cluster with a more complex genetic architecture. This locus encoded additional enzymes involved in sugar precursor biosynthesis, including dTDP-glucose 4,6-dehydratase, dTDP-4-dehydrorhamnose reductase, and dTDP-4-dehydrorhamnose 3,5-epimerase. These enzymes may broaden the repertoire of activated sugars available for EPS biosynthesis, potentially allowing the production of polysaccharides with greater structural diversity (Schmid et al., 2015). The presence of a complete phosphoregulatory module together with the priming glycosyltransferase, w*zx* flippase gene, and w*zy* polymerase gene further supports the functionality of this second locus as an independent EPS biosynthetic cluster.

Overall, these results indicate that draft genomes generated exclusively from short-read sequencing are sufficient to infer the functional potential of *L. plantarum*, as the predicted metabolic and functional repertoires were largely comparable to those obtained from the hybrid assembly. From a biological perspective, the three isolates displayed the characteristic metabolic versatility of *L. plantarum*, particularly regarding carbohydrate utilization. Together with the predicted production of plantaricins and the presence of complete EPS biosynthesis loci, these features suggest a strong capacity to colonize and persist in the table olive fermentation environment. Such traits are likely advantageous in a habitat characterized by low concentrations of fermentable carbohydrates and intense microbial competition, where both antimicrobial activity and biofilm formation may contribute to ecological fitness and persistence on the olive surface (Botta and Cocolin, 2012). Collectively, these genomic features highlight the technological potential of the three isolates for table olive fermentation and support their future evaluation as candidates with probiotic potential.

CRISPR-Cas systems constitute one of the best-characterized adaptive defense mechanisms in prokaryotes, providing protection against invading genetic elements such as bacteriophages and plasmids. Interestingly, CRISPR-Cas systems were almost completely absent from the 3 *L. plantarum* genomes analyzed in this study, with only a single incomplete type I cas locus identified in strain B282. Similar observations have been reported in other *L. plantarum* genomes, suggesting that the absence or degeneration of CRISPR-Cas systems may be relatively common within this species (Pei et al., 2021). However, the lack of CRISPR-Cas immunity does not necessarily imply increased susceptibility to foreign DNA, as bacteria frequently rely on alternative defense mechanisms. Consistent with this hypothesis, all three strains encoded additional defense systems targeting exogenous genetic elements. Strain B282 exhibited the most diverse defensive repertoire, harboring five distinct defense systems, whereas strains Lpl15 and LPT703 encoded one and three systems, respectively. Among these, AbiD, BREX, CBASS, MazEF, restriction–modification (RM), and Septu systems were identified. Although these systems operate through different molecular mechanisms, they share the common function of limiting phage propagation and restricting the establishment of foreign DNA within the bacterial cell (Tesson et al., 2022). Their coexistence within the genomes analyzed here suggests that *L. plantarum* employs a multilayered defense strategy that extends beyond CRISPR-Cas immunity. The diversity of defense systems identified among strains further highlights the remarkable genomic plasticity of *L. plantarum*. This variability may reflect differences in the evolutionary history and phage exposure of individual strains, as defense systems are frequently gained, lost, or reshuffled through horizontal gene transfer.

MGEs comprise a diverse collection of genetic components, including plasmids, genomic islands (GIs), insertion sequences (ISs), integrons, and prophages. Among these elements, plasmids are of particular interest because they frequently carry genes that confer adaptive advantages, including metabolic functions, exopolysaccharide biosynthesis, or stress tolerance (Van Kranenburg and De Vos, 1998; Raethong et al., 2022; Marín-Gordillo et al., 2026). In the present study, plasmid content could only be confidently resolved in strain Lpl15, whose hybrid assembly revealed the presence of three plasmids. In contrast, the fragmented assemblies of strains B282 and LPT703 prevented reliable estimation of plasmid number and structure. Comparative BLAST analyses against the NCBI plasmid database indicated that the two largest plasmids shared the highest similarity with plasmids previously identified in *L. plantarum* strains, whereas the smallest plasmid exhibited greater similarity to plasmids from species belonging to the genus *Lentilactobacillus*, suggesting a possible history of horizontal gene transfer between closely related lactic acid bacteria. GIs represent another important component of the accessory genome and are frequently associated with the acquisition of adaptive traits (Juhas et al., 2009). However, similarly to plasmids, accurate identification of genomic islands was strongly influenced by assembly quality. In the draft genomes of B282 and LPT703 strains, it was not possible to confidently discriminate between genomic islands and plasmid-derived regions due to assembly fragmentation. Consequently, only the genomic islands identified in the complete Lpl15 genome could be interpreted with confidence. These regions encoded functions potentially associated with environmental adaptation, including an Opp oligopeptide transport system, genes related to oxidative metabolism, and carbohydrate utilization functions. Such functions may contribute to nutrient acquisition and metabolic flexibility in complex fermentation environments (Juhas et al., 2009). ISs constitute one of the most abundant classes of mobile genetic elements in bacterial genomes and are important drivers of genome rearrangement and evolution (Broadbent et al., 2013). The hybrid assembly of strain Lpl15 contained 16 predicted IS elements, whereas only five and seven ISs were identified in strains B282 and LPT703, respectively. Several factors may explain this difference. First, Lpl15 was shown to harbor three plasmids, which are frequently enriched in insertion sequences. Similar observations have been reported in other members of the genus *Lactiplantibacillus*. For example, Marín-Gordillo et al. (2026) identified 133 IS elements in *L. pentosus* LP309, a strain containing eight plasmids. Second, the repetitive nature of IS elements makes them particularly difficult to resolve using short-read sequencing data, often leading to collapsed or fragmented assemblies (Peng et al., 2021). Consequently, the lower number of ISs detected in B282 and LPT703 may reflect technical limitations rather than genuine biological differences. During the lysogenic cycle, bacteriophages integrate into the bacterial chromosome and persist as prophages (Moineau, 2013). Such elements are commonly found in members of the family Lactobacillaceae, including *L. plantarum*. The number of prophages identified in the present study was comparable to that reported for other *L. plantarum* genomes (Tenea and Ortega, 2021; Aziz et al., 2022). Strains B282 and Lpl15 each harbored three intact prophages, whereas only a single prophage was detected in strain LPT703. Beyond their role as remnants of past phage infections, prophages can contribute to bacterial evolution through the introduction of novel genetic material and the modulation of host physiology (Brüssow et al., 2004).

Overall, the comparison between draft and hybrid assemblies highlights important differences in the resolution of mobile genetic elements. Whereas defense systems could be reliably identified regardless of assembly strategy, accurate characterization of plasmids, GIs, and ISs was strongly dependent on assembly contiguity. The repetitive nature of these elements and their frequent occurrence at assembly breakpoints complicate their reconstruction from short-read data alone. In contrast, prophage prediction appeared to be less affected by assembly fragmentation. This observation may be explained by the fact that prophage regions frequently occur as relatively large and contiguous chromosomal regions, making them easier to reconstruct from short-read assemblies. These observations emphasize the value of hybrid sequencing approaches for comprehensive characterization of the accessory genome and mobilome of *L. plantarum*.

*Lactiplantibacillus plantarum* currently holds Qualified Presumption of Safety (QPS) status from the European Food Safety Authority (EFSA), and the safety of members of the genus has been repeatedly demonstrated in the literature (Hummel et al., 2007; Benítez-Cabello et al., 2019). Nevertheless, antimicrobial resistance remains an important aspect to consider when evaluating strains intended for food or biotechnological applications. Previous studies have reported the presence of acquired resistance determinants in some lactobacilli, including genes conferring resistance to chloramphenicol, tetracyclines, β-lactams, and aminoglycosides (Sharma et al., 2014). In the present study, the only resistance determinant identified corresponded to vancomycin resistance. However, resistance to vancomycin is considered an intrinsic and non-transferable trait in lactobacilli and therefore does not represent a safety concern according to current EFSA guidelines. Together with the absence of virulence-associated genes, these findings further support the safety of the three isolates analyzed in this work.

In recent years, Average Nucleotide Identity (ANI) has become the genomic standard for bacterial taxonomic classification, largely replacing DNA–DNA hybridization (DDH) approaches. ANI thresholds of approximately 95% are widely accepted for species delineation. The ANI analysis performed in this study included 1,820 pairwise genome comparisons and confirmed the assignment of all strains to the species *L. plantarum*, with values ranging from 98.25% to 99.99%. Although clusters of relatively high and low genomic similarity were observed, no clear relationship between ANI values and ecological origin was detected. These results indicate a high degree of genomic conservation across strains isolated from distinct environments, including vegetable fermentations, dairy products, meat-derived foods, and human microbiota. The pangenome analysis identified a total of 6,480 gene clusters, comprising 2,351 core gene clusters and 4,129 accessory gene clusters. The large proportion of accessory genes highlights the remarkable genomic flexibility of *L. plantarum* and is consistent with its ability to colonize a wide variety of ecological niches (Siezen et al., 2010). Similar observations have been reported previously and support the concept of *L. plantarum* as a nomadic species characterized by a versatile lifestyle and broad metabolic repertoire (Sato et al., 2024). Rather than representing dispensable genetic material, many accessory genes may provide selective advantages under specific environmental conditions, facilitating adaptation to diverse habitats and fluctuating ecological pressures. The ANI and phylogenomic analyses revealed a high degree of conservation among *L. plantarum* genomes, with no clear clustering according to ecological origin. Combined with the substantial size of the accessory genome, these findings suggest that ecological diversification within the species is associated mainly with variation in accessory genetic content rather than with divergence of the core genome. One limitation of this study is the use of fragmented draft genomes generated from short-read sequencing, which may have led to an underestimation of coding sequences, particularly those associated with the mobilome, as demonstrated by our comparison with the hybrid assemblies.

The KEGG provides a valuable framework for linking genomic information to metabolic pathways and cellular functions. In the present study, pathway completeness analysis revealed that amino acid biosynthesis, mixed-acid fermentation, transport systems, and carbohydrate storage pathways were among the most complete metabolic categories across the 35 *L. plantarum* genomes. In contrast, pathways associated with secretion/competence and carbon fixation showed the highest levels of incompleteness. Hierarchical clustering based on pathway completeness profiles identified two major genome groups. One cluster was predominantly composed of strains isolated from vegetable fermentations (11 of 16 genomes), whereas the second cluster mainly contained strains of animal-associated origin (14 of 19 genomes). The most notable difference between these groups was the absence of complete nitrogen metabolism pathways, specifically nitrite oxidation and dissimilatory nitrate reduction, in the cluster enriched with vegetable-associated isolates. Although the ecological significance of this pattern remains unclear, the differential distribution of these pathways may reflect niche-dependent selective pressures acting on nitrogen metabolism. However, experimental studies would be required to determine whether these genomic differences translate into functional adaptations (Martino et al., 2016).

The remarkable ecological breadth of *L. plantarum* is closely linked to its ability to utilize a wide variety of carbohydrate substrates. This versatility is particularly relevant because the species inhabits environments with highly diverse carbohydrate compositions, ranging from plant tissues and fermented vegetables to animal-derived products and host-associated niches. CAZymes play a central role in this process by catalyzing the synthesis, modification, and degradation of complex carbohydrates. Hierarchical clustering of CAZyme presence–absence profiles identified three major genome clusters, each displaying a predominant ecological origin rather than representing strictly niche-specific groups. The first cluster, composed predominantly of strains isolated from vegetable fermentations (8 of 11 genomes), was significantly enriched in the glycosyltransferase family GT125. Members of this family are associated with exopolysaccharide biosynthesis, suggesting an increased genetic potential for EPS production among plant-associated strains. This observation is particularly relevant for table olive fermentations, where biofilm formation on the fruit surface has been extensively documented and may contribute to bacterial persistence under fermentation conditions (López-García et al., 2023b). The second cluster, primarily composed of strains from animal-associated sources (8 of 10 genomes), was significantly enriched in GH31_15, a glycoside hydrolase family annotated as α-glucosidase. Enzymes belonging to this family are involved in the hydrolysis of O-glycosyl compounds and may contribute to the utilization of more specialized carbohydrate substrates encountered in animal-associated environments. The third cluster exhibited a heterogeneous ecological composition, including both plant- and animal-associated isolates, and was enriched in GH20, GH92, and GH125. These families encode enzymes such as N-acetyl-β-hexosaminidase, α-1,2-mannosidase, and α-1,6-mannosidase, respectively, which are involved in the degradation of structurally complex glycans. The enrichment of these families may therefore reflect a broader metabolic capacity to exploit diverse carbohydrate sources. Taken together, both the KEGG and CAZyme analyses suggest the existence of functional trends associated with ecological origin. However, these patterns were not sufficient to establish niche-specific signatures or to clearly assign genomes to a particular habitat. Instead, the observed differences appear to represent gradual shifts in metabolic potential between groups of strains associated with plant- and animal-derived environments. These findings are consistent with the concept of *L. plantarum* as a highly versatile species whose ecological diversification is driven by flexible functional repertoires rather than by strict niche specialization (Siezen et al., 2010; Martino et al., 2016).

Protein domains frequently constitute the functional units responsible for specific biological activities and therefore provide valuable information for understanding genome diversification and ecological adaptation. The Pfam database is one of the most widely used resources for the annotation of protein families and domains and has been extensively employed to investigate functional differences among bacterial genomes. Consequently, analysis of Pfam domain content may provide an alternative perspective for exploring ecological differentiation beyond conventional phylogenetic approaches. Principal Component Analysis (PCA) based on these domains explained approximately 30% of the total variance within the first two components. Although genomes from different ecological origins showed substantial overlap, strains isolated from meat products and cucumber fermentation tended to cluster more closely together than genomes from other sources, indicating that ecological origin explains only a limited proportion of the observed functional variation. Interestingly, the highest-ranking domains were predominantly related to defense systems, mobile genetic elements, prophages, and restriction–modification systems (Figure 8). These results are consistent with the previous analyses of defense mechanisms and mobilome composition, which also revealed substantial variability among genomes despite the high conservation of the core genome (Siezen et al., 2010; Martino et al., 2016; Duar et al., 2017). Previous studies have highlighted the importance of mobile genetic elements as drivers of bacterial diversification. In some cases, prophage content alone has been shown to discriminate among closely related lactobacilli (Pei et al., 2021), while other studies have demonstrated that mobilome-associated genes contribute substantially to genome plasticity and ecological adaptation (Patrick et al., 1994; De Las Rivas et al., 2005). The predominance of mobilome- and defense-related domains among the most informative Pfam features identified by Random Forest suggests that these genomic regions may play a central role in shaping the functional diversity of *L. plantarum* populations.

## 5 Conclusion

Short-read assemblies provided functional annotations comparable to those obtained from hybrid assemblies, although long-read sequencing substantially improved the resolution of repetitive regions and enabled a more accurate characterization of the *L. plantarum* mobilome, including plasmids, genomic islands, and insertion sequences. Comparative genomic analyses revealed a highly conserved core genome across strains from different ecological origins, with no evidence of niche-specific phylogenomic lineages. In contrast, differences associated with ecological origin were primarily observed within the accessory genome, particularly in metabolic functions, CAZyme repertoires, defense systems, and mobile genetic elements. Together, these findings further support the view that ecological differentiation in *L. plantarum* is largely driven by variation within the accessory genome rather than by divergence of the core genome. Moreover, the genomic features identified in the three newly sequenced strains provide a valuable foundation for future studies exploring their technological and probiotic potential.

## Data Availability

The assembled genome sequences and the corresponding raw sequencing reads are publicly available in the European Nucleotide Archive (ENA) under BioProject accession number PRJEB111681.
